# A multi-decade dataset of monthly beach profile surveys and inshore wave forcing at Narrabeen, Australia

**DOI:** 10.1038/sdata.2016.24

**Published:** 2016-04-12

**Authors:** Ian L. Turner, Mitchell D. Harley, Andrew D. Short, Joshua A. Simmons, Melissa A. Bracs, Matthew S. Phillips, Kristen D. Splinter

**Affiliations:** 1 Water Research Laboratory, School of Civil and Environmental Engineering, UNSW Australia, Sydney, NSW 2052, Australia; 2 School of Geosciences, University of Sydney, NSW 2006, Australia

**Keywords:** Civil engineering, Physical oceanography, Geomorphology

## Abstract

Long-term observational datasets that record and quantify variability, changes and trends in beach morphology at sandy coastlines together with the accompanying wave climate are rare. A monthly beach profile survey program commenced in April 1976 at Narrabeen located on Sydney’s Northern Beaches in southeast Australia is one of just a handful of sites worldwide where on-going and uninterrupted beach monitoring now spans multiple decades. With the Narrabeen survey program reaching its 40-year milestone in April 2016, it is timely that free and unrestricted use of these data be facilitated to support the next advances in beach erosion-recovery modelling. The archived dataset detailed here includes the monthly subaerial profiles, available bathymetry for each survey transect extending seawards to 20 m water depth, and time-series of ocean astronomical tide and inshore wave forcing at 10 m water depths, the latter corresponding to the location of individual survey transects. In addition, on-going access to the results of the continuing monthly survey program is described.

## Background & Summary

**Figure 1:**
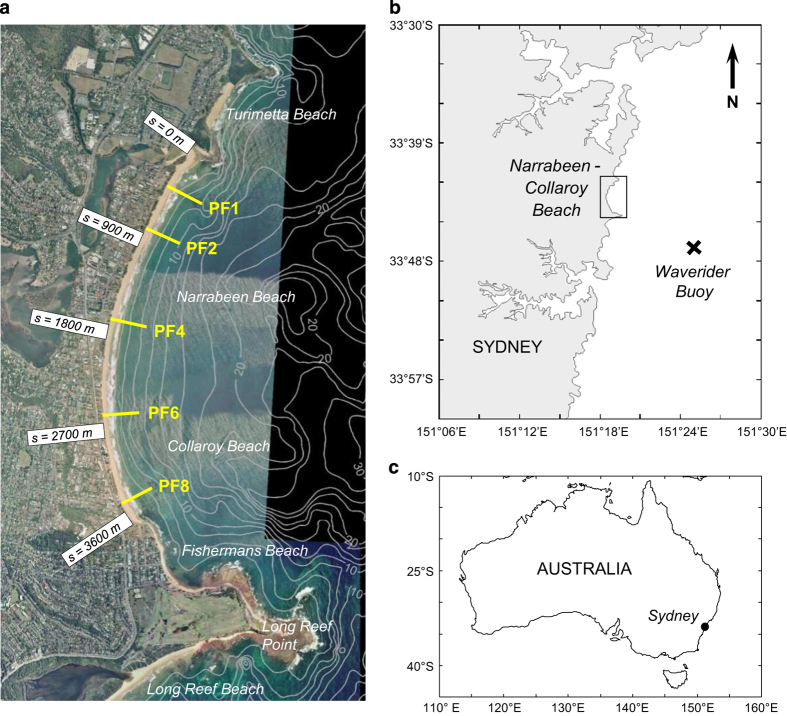
(**a**) Aerial photo (source: NSW Department of Lands) of the Narrabeen embayment—showing the locations of the five monthly survey transects (PF1, PF2, PF4, PF6, and PF8), depth contours at 2.5 m intervals, and the local alongshore coordinate system relative to the northern headland; (**b**) the beach with respect to the Sydney coastline and the location of the Sydney waverider buoy; (**c**) map of Australia (adapted from ref. 35).

Long-term datasets that record and quantify the variability, changes and trends in morphology observed at sandy beaches are rare. A monthly beach profile survey program that commenced in April 1976 at Narrabeen located on Sydney’s Northern Beaches in southeast Australia (Fig. 1) is one of a limited number of sites globally where on-going and uninterrupted beach monitoring now spans multiple decades and the use of these data has been reported in the published literature (Table 1; refs 1–16).

In the 1970s and 1980s the growing database of beach surveys at Narrabeen was key to the pioneering work by Australian coastal geomorphologists that resulted in the formulation of the Morphodynamic Beach State Model^17^, which today remains the standard classification scheme used by coastal scientists worldwide to describe different natural sandy beach states, their characteristic morphodynamic process signatures and associated wave and sediment environmental controls^17–27^. During the 1990s related studies at Narrabeen included a focus on surfzone rip currents and the emergence of new insight to the associated hazards to beach swimmers^28,29^.

At the turn of the 21st Century the record of beach changes at Narrabeen had extended to sufficient length that longer-term cycles and underlying trends in beach behaviour began to be revealed^30,31^. This prompted significant new interest in the wider use of the Narrabeen survey dataset to further identify and explore potential linkages between regional-scale climatic forcing and sandy coastline response^32–36^. At the same time, recognition within the research community that regional-scale wave climates can be expected to change and sea levels in the coming decades will continue to rise^37,38^ helped strengthen the awareness of the fundamental importance of sustained coastline monitoring programs. In particular, the monthly observations from Narrabeen provided an all-too-rare data resource to calibrate and test new models aimed at developing better prediction tools of present and future variability and changes along sandy coastline worldwide^39–47^.

The monthly beach profile survey program at Narrabeen will reach its 40-year milestone in April 2016. It is therefore timely to facilitate, through publication of the dataset, the unrestricted use of this resource by as wide a cross-section of the coastal research community as possible. The complete archived dataset described and detailed here includes the monthly subaerial profiles, bathymetries and time-series of astronomical tide and offshore wave forcing transformed to the inshore location corresponding to each of the individual survey transects.

It is envisaged that open and easy access to these data may provide a new stimulus to coastal morphodynamic modellers worldwide to develop, test and (it is hoped) significantly advance the next generation of beach erosion-recovery hindcasting and forecasting tools. As a discipline, our ability to predict anticipated coastal changes in the context of a changing climate is presently in its relative infancy. In the meantime, as the current custodians of this valuable resource it is the authors’ intention that collection of monthly profile data at Narrabeen will continue for the foreseeable future, as our contribution to generations who follow. In addition to the complete and archived dataset that accompanies this publication, access details are also provided to a ‘live’ online Narrabeen monitoring program repository, where on-going monthly profile surveys will continue to be updated.

## Methods

The full archived dataset comprises:

- Monthly cross-shore subaerial profile surveys at the five locations where these have been continued from 1976 up to the present time;
- Cross-shore bathymetry transects for each of the five survey profile lines extending to 20 m water depth;
- Hourly local inshore significant wave height, peak wave period and mean wave direction (Hs,Tp, Dir) at 10 m water depth immediately seawards of each of the five survey transects, derived from the transformation of measured deepwater waves and gap-filled using a newly-available wave hindcast; and
- Astronomical tide at a frequency of 15 min.

To coincide with this publication, and in addition to the accompanying archived dataset spanning 1976–2016 [Data Citation 1], unrestricted access is now also available to an online ‘live’ data repository located at http://narrabeen.wrl.unsw.edu.au containing additional information and resources, including: all the historical as well as newly acquired profile surveys updated each month, a range of survey data visualisation tools; the lookup table (MATLAB software code) used to transform deep to inshore waves specific to the location of each survey transect, and the contact details to external organisations (to which the authors have no affiliation) where additional wave and water-level information may be requested.

### Site description

The coastline of southeastern Australia includes over 700 embayed sandy beaches averaging 1.3 km in length separated by rocky headlands^48^. The 3.6 km-long Narrabeen-Collaroy embayment (hereafter simply referred to as ‘Narrabeen’) is situated within the Northern Beaches region of metropolitan Sydney. Locally, the sandy beach that spans the entire embayment is referred to as Narrabeen beach towards the north and Collaroy beach in the south, with the small section of beach adjacent to the prominent headland at the extreme southern end called Fishermans beach (Fig. 1).

The beach sediments at Narrabeen were deposited as a regressive barrier in the mid-Holocene approximately 300 m landward of the present-day shoreline. The barrier subsequently prograded through a series of foredune ridges, with the most seaward ridge of the modern embayment dated at 3 ka. The granulometry is approximately uniform along the beach and characterized by fine to medium quartz sand (D_50_≅0.3 mm) with ~30% carbonate fragments. A lagoon now backs the northern half of the barrier and is connected to the ocean via a shallow narrow (~50 m-wide) inlet at the embayment’s northern extremity that intermittently opens and closes to the ocean^49^.

The adjacent headlands and curvature of the embayment result in a distinctive alongshore wave energy gradient. Dissipative-intermediate beach conditions typically prevail in the north, transitioning to lower energy and intermediate-reflective beach conditions towards the south. The northern end of Narrabeen is characterised by single-bar rhythmic bar-beach (RBB) to transverse bar-rip (TBR) intermediate beach states and a subaerial berm that varies up to 80 m in width, backed by a vegetated foredune up to 9 m in height above mean sea level (MSL). At the southern end of the embayment urban development has encroached on to much of the foredunes, which reach only 3 to 4 m in height, and the beach consists of a berm that varies up to 60 m in width and a single-bar system that tends towards the lower-energy low-tide terrace (LTT) and reflective beach states. Tides are microtidal and semidiurnal with a mean spring tidal range of 1.3 m.

The deepwater wave climate for the Sydney region is of moderate to high wave energy (mean Hs≅1.6 m and Tp≅10 s) and dominated by persistent long period swell waves from a SSE direction. These swell waves are generated from mid-latitude cyclones that propagate approximately 5–9 times per month across the southern Tasman Sea, south of mainland Australia^50^. Superimposed on these background swell waves are storm events that are typically defined for this region by a significant wave height threshold of 3 m, corresponding to the 0.95 quantile^51^. These storm waves are derived from a number of sources and directions: tropical cyclones from the northeast, east-coast lows from the east and intensified mid-latitude cyclones from the south. The wave climate exhibits a mild seasonal cycle, with high-energy mid-latitude cyclones and east-coast lows more prominent in the Austral winter months and low-energy short-period seas derived from local north-easterly sea-breezes more prominent in the Austral summer^51^. At inter-annual time scales, the wave climate is influenced by the El Niño/Southern Oscillation (ENSO), with La Niña periods typically having a more energetic and easterly wave climate and El Niño periods a less energetic and more southerly wave climate^32,33,36^. The distinct wave energy gradient in the local shallow-water wave climate at Narrabeen is to a large degree a result of the sheltering of southerly waves by the 1.5 km Long Reef Point headland that forms the embayment’s southern extremity. Numerical wave modeling^33^ indicates breaking wave height is approximately 30% higher at the northern end compared to the southern end for average wave conditions. This situation is reversed, however, for northeast waves, with breaking wave heights approximately 30% larger in the south relative to the north. An equivalent reversal in the wave angle of incidence is also observed, with southerly waves resulting in northerly-directed alongshore currents and northeast waves resulting in southerly-directed alongshore currents.

### Monthly beach profile surveys and bathymetry transects

The beach monitoring program at Narrabeen can be divided into two distinct periods: the first three decades when a simple and traditional survey technique was employed; and from 2004 onwards when the monitoring program was significantly expanded, and the use of new and emerging survey technologies have been progressively implemented. A full history of the beach monitoring program at Narrabeen is detailed in refs 52,53. A time-line and summary of the various survey methods employed is presented below.

### 1976–2006: historical profile surveys

The years between 1976 and 2006 constitute the period of conventional profile line surveys undertaken by Professor Andrew Short and volunteers of the Coastal Studies Unit, University of Sydney employing the Emery method^54^. This simple, rapid and low-cost technique uses a measuring tape for cross-shore distance, and vertical elevation changes are calculated using line-of-sight between two graded rods and the horizon. Commencing with the landward rod on a fixed benchmark, the distance between the two rods is first measured using the measuring tape. The change in elevation between the two rods is then calculated by using the line-of-sight with the horizon and markings on the rods as a reference. This process is repeated at each subsequent measurement point along the entire length of the cross-shore profile line. A detailed validation of these historical survey data is provided in the ‘Technical Validation’ section.

The monitoring program during the 1970s based on this use of the Emery method initially comprised fortnightly cross-shore profile surveys at a total of fourteen profile lines along the embayment. Each profile line was surveyed at spring low tide from a fixed benchmark located in the stable dune area down to a swimming depth within (and sometimes beyond) the surf zone. This labour-intensive approach typically extended each surveyed profile to depths of 1–4 m below mean sea level, depending on the prevailing surf conditions. The cross-shore spacing of each measurement was 10 m.

Following the first few years of these fortnightly surveys at fourteen profile lines, a pragmatic decision was made to reduce and focus on-going effort to achieve monthly surveys at a lesser number of five representative profile lines. These five profiles that continue to the present day are numbered 1, 2, 4, 6 and 8 (north to south, hereafter identified as PF1, PF2, PF4, PF6 and PF8) and their locations are indicated in Fig. 1; their non-sequential numbering corresponds to the original numbering scheme when 14 profiles were surveyed. The seaward survey limit for each of these five representative profiles was also changed to the more easily achievable intersection with mean sea level (i.e., approximately wading depths). These early and pragmatic decisions to limit the number and cross-shore extent of each profile line are undoubtedly the key reasons why monthly surveys were subsequently achieved by the same personnel during the ensuing three decades.

### 2004-present: new survey technologies

Recognising the unique and growing value of the Narrabeen survey dataset worldwide, beginning in 2004 efforts were initiated by the UNSW Water Research Laboratory^55^ to secure, improve and expand the monitoring program into the future through the use of new survey technologies. This commenced in July 2004, with the decision to transition the historical profile line surveys from the Emery method to the use of high-accuracy RTK-GPS technology (vertical accuracy≈±0.03 cm). Following a 16-month validation period during which surveys were undertaken concurrently using the Emery method and RTK-GPS (refer ‘Technical Validation’ section), the use of RTK-GPS as the standard survey method for the five profile lines was adopted in May 2005. The cross-shore resolution of each profile survey was also increased at this time from the original 10 m measurement spacing to near-continuous (i.e., approximately every 0.10 m cross-shore). At the same time as the use of RTK-GPS was implemented in early 2004, an Argus coastal imaging station^56^ was installed atop the 44 m high Flight Deck apartment building at South Narrabeen. Since this time, this station has continuously collected hourly daylight images of the southern sector of the beach from five separate cameras (the field of view encompassing PF6 and PF8). These images are available for public viewing and download (http://ci.wrl.unsw.edu.au). Since 2004 several additional survey techniques have been progressively implemented at Narrabeen (Table 2) to complement the on-going monthly profile surveys detailed here, with the objective to begin to build for the future an expanded dataset with which to gain greater understanding of beach morphodynamics at more detailed spatial and temporal resolutions. It is our goal that, as the necessary resources to undertake rigorous QA, data archiving, online storage and delivery become available, open access to these additional data will be facilitated via the ‘live’ data repository at: http://narrabeen.wrl.unsw.edu.au.

Cross-shore bathymetry transects to accompany the historical profile surveys have been obtained from 11 hydrographic surveys conducted by the NSW Office of Environment and Heritage (OEH) during the period 2011–2015. These hydrographic surveys have been undertaken using a variety of methods: a single-beam jetski-mounted system for shallow water depth soundings (8 jetski surveys in total); a single-beam boat-mounted system for shallow-intermediate water depth soundings (2 surveys); and a multi-beam boat-mounted system for intermediate water depth soundings at high resolution (1 survey).

### Waves and astronomical tides

Hourly offshore wave measurements of the significant wave height Hs, peak wave period Tp and mean wave direction Dir have been recorded by the Sydney directional waverider buoy since March 1992, located 11 km offshore of Narrabeen (33° 47′S, 151° 25′E) in 80 m water depth (Fig. 1). In order to obtain a continuous directional wave time-series spanning as much as is currently possible of the full duration of the beach survey program, wave data prior to 1992 as well as gaps in the wave measurement record (typically a few days, in total 5%) have been filled by hourly hindcast waves. A high-resolution wave hindcast dataset (approx. 7 km grid spacing) developed by the Centre for Australian Weather and Climate Research (CAWCR)^57^ that currently spans the period 1979–2014 at the closest grid point to Sydney (33° 48′S, 151° 24′E) was used for this purpose. The resulting continuous (hourly) offshore wave time-series from January 1979 to October 2014 was then transformed to 10 m water depths at the location of each of the five cross-shore profile lines using the nearshore wave model SWAN^58^. The SWAN model transformation (refer below for technical validation) was undertaken by means of a lookup table based on 1573 model run combinations of offshore Hs, Tp and Dir. Physical processes activated in the SWAN model include triad wave-wave interactions, wave growth, white-capping, depth-induced wave breaking and bottom friction (adopting default parameters for each process). A JONSWAP wave spectrum was assumed with a peak enhancement factor of 3.3 (default). These discrete wave combinations used for SWAN model runs span the range of offshore wave conditions along this coastline, from significant wave heights between 0.25 and 9.5 m, wave periods between 2 and 17 s and wave directions between 0° and 245° TN. Since wave period is unchanged in shallow water, peak wave periods at the 10 m water depth locations are assumed equal to offshore values (i.e., no wave period transformation is undertaken).

Astronomical Tides over the same time period as the combined wave data (i.e., January 1979–October 2014) have been derived at 15 min intervals using the tidal analysis package T_Tide^59^ based on available water-level data (1987–2012) at the nearby HMAS Penguin tide gauge (33° 49′31.66″S, 151° 15′30.71″E). This subset of measured water-level data was obtained from an external organisation (contact details provided in online ‘live’ data repository http://narrabeen.wrl.unsw.edu.au). Absolute tidal anomalies due to short-term atmospheric and oceanographic fluctuations for this 25 year time period are found to be limited to 0.19 m for 95% of the time and 0.26 m for 99% of the time. A comprehensive analysis and discussion of historical and recent sea-level trends and variability in this region are detailed in ref. 60.

### Code availability

The lookup table used to transform the combined deepwater directional wave data to the 10 m water depth corresponding to the inshore location of each of the five individual profile survey transects is available at http://narrabeen.wrl.unsw.edu.au. This is implemented as a single and fully commented MATLAB function.

The T_Tide software code used to generate the Astronomical Tide time-series for Sydney that is provided with this dataset is freely available at: https://www.eoas.ubc.ca/~rich/#T_Tide. Output statistics from this T_Tide analysis for Sydney, including all significant tidal amplitudes, is also available at http://narrabeen.wrl.unsw.edu.au.

## Data Records

The full archived dataset can be obtained at [Data Citation 1]. Access to these same data plus additional background information, online visualisation tools and the profile surveys as they continue to be updated on a monthly basis are also provided at http://narrabeen.wrl.unsw.edu.au.

**Figure 2:**
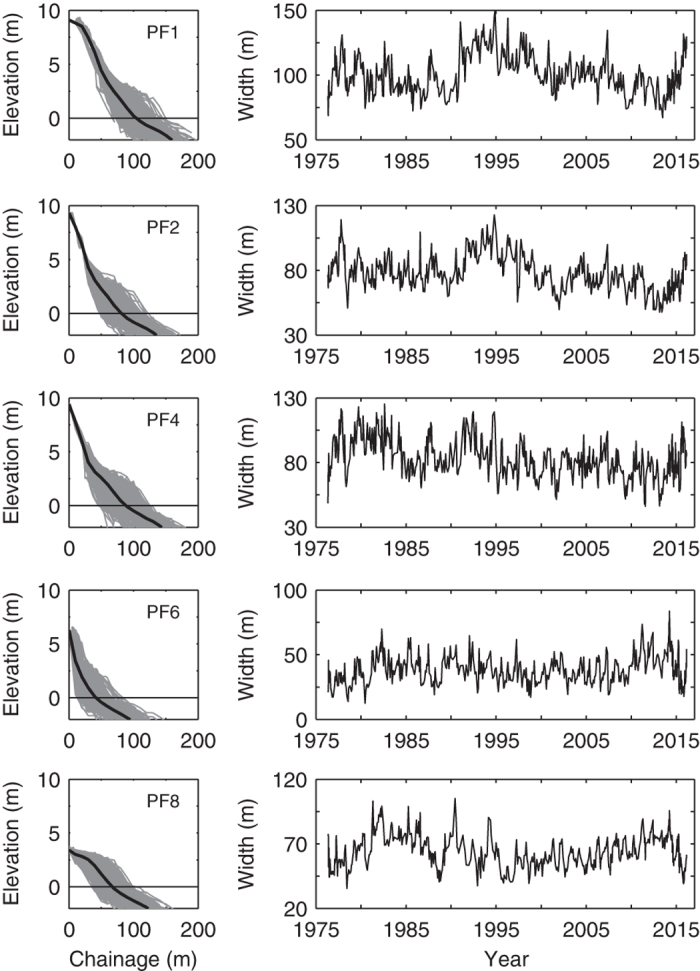
Narrabeen cross-shore profile surveys (April 1976–February 2016). Left panels show all monthly beach profiles (mean profile in bold); right panels show corresponding time-series of beach width at the 0 m AHD (Australian height datum) contour elevation.

Table 3 documents the data format and metadata for the complete monthly beach profile dataset April 1976–February 2016. Figure 2 shows graphically the mean profile and envelope of profile change that has been recorded at each survey profile PF1, PF2, PF4, PF6 and PF8. Also shown in this figure is the time-series of beach width 1976–2016 recorded at the 0 m AHD (Australian Height Datum) contour elevation, corresponding to (approximately) mean sea level.

The five profiles lines are provided in a single comma-delimited text file ‘Narrabeen_Profiles.csv’. Note that survey measurements prior to May 2005 (survey method ‘EMERY’ in column 5) were undertaken at a fixed cross-shore spacing of 10 m (no interpolation), while surveys undertaken after this time (survey method ‘GPS’) have been interpolated to a standard 1 m cross-shore spacing. Intermittent survey measurements in the stable dune areas of each profile have been carried through to the following survey date and are identified by the flag ‘DUNEFILL’ in column 5.

**Figure 3:**
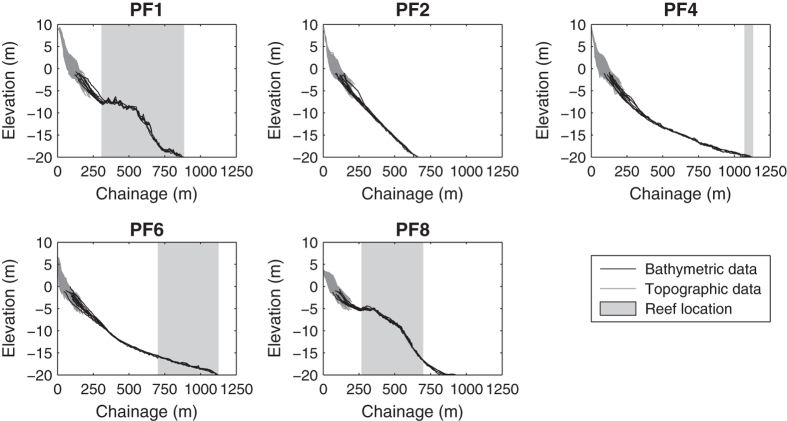
Bathymetry transect data corresponding to the five profile lines. Bathymetry data are derived from 11 hydrographic surveys of the entire embayment undertaken intermittently between 2011 and 2015 by the NSW Office of Environment and Heritage. Topographic data (the monthly beach profile surveys) are also indicated for reference.

Table 4 documents the specific data format and metadata for the bathymetry transects extending seawards from approximately −2 to −20 m AHD, corresponding to the location of each of the five beach profile lines. These data are provided in a single comma-delimited text file ‘Narrabeen_Bathymetry.csv’. Bathymetry transect data (Fig. 3) were obtained from 11 intermittent hydrographic surveys conducted between 2011 and 2015. The data is provided at a fixed 1 m cross-shore spacing referenced to the same origin as the profile line surveys. Any rock (i.e., non-erosive) reefs located along the bathymetry transects (most notably in profiles PF1 and PF8) are identified in column 5 by ‘REEF’, whereas sandy bed types are labelled ‘SAND’. Single-beam jetski-mounted measurements are denoted in column 6 by the flag ‘SBEAMJETSKI’, single-beam boat mounted surveys by ‘SBEAMBOAT’ and multi-beam boat-mounted surveys by ‘MBEAMBOAT’.

**Figure 4:**
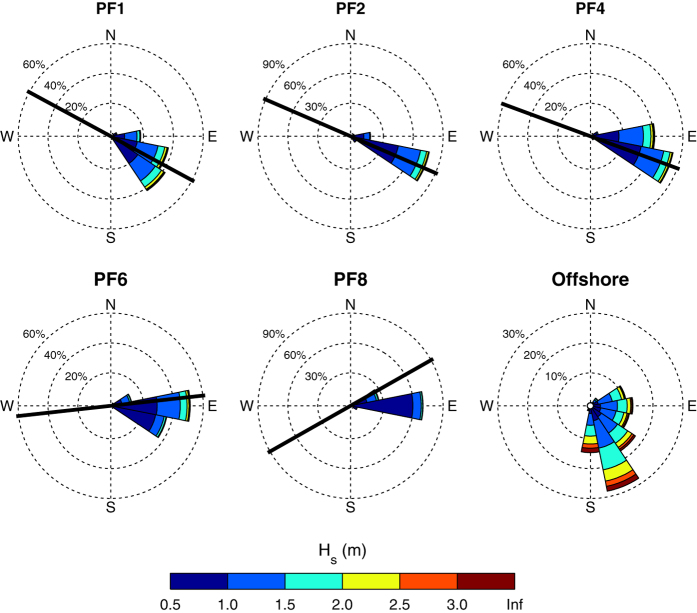
Wave roses for the Sydney deepwater wave climate as well as the five inshore wave datasets located at 10 m water depths for the five survey transects. Deepwater wave data are based on combined wave hindcast/measured waves between 1979 and 2014. Inshore wave data are derived from a SWAN model transformation. Profile line orientations are indicated by a solid black line. Note the differing axis scaling for individual waves roses.

Table 5 documents the formats and metadata for the hourly time-series of inshore significant wave height (Hs), peak wave period (Tp) and mean wave direction (Dir) at 10 m water depth directly seaward of each of the five beach profile lines. The comma-delimited text file is called ‘Inshore_Waves.csv’. The wave roses shown in Fig. 4 summarise the long-term features of the deepwater and inshore wave climate for this same period, showing the dominance of persistent long period swell waves from a SSE direction. Note that these wave time-series start approximately 3.5 years after the commencement of the beach survey program and presently end in October 2014. At the time of writing no source of deepwater wave information (source identified in column 5 as ‘MEAS’—measured, ‘HIND’—hindcast, or ‘INTERP’—linear gap-interpolations) outside of this ~35 year time-window was available.

Table 6 documents the comma-delimited ‘Astronomical_Tide.csv’ text file, format and metadata for the hourly time-series of astronomical tide spanning the identical period of the inshore wave time-series.

## Technical Validation

### Emery method survey validation

Validation of the Emery method surveys was assessed over a 16-month period (May 2005–August 2006) when all five profile lines were measured concurrently by both the Emery method and high-accuracy RTK-GPS. Comparing the complete profiles derived from the two survey methods (80 profiles in total), vertical deviations were found to be approximately normally distributed with a mean of −0.03 m and a standard deviation of 0.13 m. In the intertidal zone (between 0 and +2 m AHD), this translates to a cross-shore standard deviation of 1.1 m. Considering the magnitude of cross-shore variability at this site (standard deviation of width at the 0 m AHD contour=11–14 m), this represents a signal-to-noise ratio (SNR) in the order of 10:1. A full summary of the Emery method validation and SNR analyses at various time scales for the Narrabeen beach profile data set is detailed in ref. 52.

### CAWCR wave hindcast data validation

The CAWCR wave hindcast dataset has been validated at both a global level (using global satellite altimeter data) and regional level (using Northern Hemisphere and South Pacific wave buoys), as documented by ref. 57. In order to assess the applicability of this wave hindcast data for the Sydney region, wave hindcast data at the nearest grid point was compared to hourly measured data from the Sydney waverider buoy (1992–2014, *n*=1,65,388). Statistics used for the validation include the Pearson correlation coefficient *R*, mean bias (mean bias=hindcast—measured data) and the root-mean-square-error (RMSE). These statistics are summarised in Table 7 for all three wave parameters (Hs, Tp and Dir) at both offshore and inshore locations.

This validation indicates a strong agreement between hindcast and measured offshore data for Hs (*R*=0.90, bias=−0.03 m, RMSE=0.43 m) that decreases in accuracy for Dir (*R*=0.70, bias=−8.4°, RMSE=30.1°) and Tp (*R*=0.51, bias=−0.62 s, RMSE=2.3 s). Such results are consistent with those observed both at a global level and with the Northern Hemisphere/South Pacific buoys described by ref. 57. The decrease in accuracy related to the peak wave period can in part be explained by the discontinuity of the measured peak wave period data, which can vary substantially under mixed sea/swell regimes and when there is bimodality in the wave spectra. When transforming both measured and hindcast data to the five inshore locations, a dampening of the wave direction bias (e.g., from −8.4° offshore to −1.5° at PF8) as well as the overall RMSE is observed. This dampening is related to an overall decrease in wave directional variability as waves are refracted and attenuated from deep to shallow water.

### Nearshore wave transformation validation

To assess the validity of the SWAN nearshore wave transformation used to transform offshore (deepwater) wave data to inshore (intermediate-shallow) values, the same lookup table was applied and compared to hourly data from an inshore waverider buoy deployed within the Narrabeen embayment spanning a four-month period between July and November 2011. The location of this buoy (33°43′17″S, 151°18′15″E) was approximately halfway between PF4 and PF6, at the same 10 m water depth. As a means of evaluating the additional uncertainty that is introduced by using offshore wave hindcast as opposed to measured data for this purpose, both measured and hindcast offshore wave data were also independently transformed and compared to inshore measurements. A total of 2,431 measurements of inshore significant wave height and direction were used for the validation. Validation statistics were the same as those described above for the hindcast validation: correlation coefficient *R*, mean bias (mean bias=transformed—measured data) and RMSE. These statistics are summarized in Table 8.

In general, both the transformed measured and transformed hindcast data indicate good agreement with inshore measured values, with correlation coefficients of 0.94 and 0.91 for measured and hindcast Hs respectively; and corresponding 0.74 and 0.62 for Dir. Mean biases for both measured and hindcast deepwater waves transformed to the nearshore are likewise small and are in the order of −0.02 and −0.04 m respectively for Hs (i.e., a slight underestimation of inshore wave heights) and 4.4° and 2.9° respectively for Dir (i.e., a slightly more southerly estimation of the wave direction). The relatively minor reduction in accuracy for the wave hindcast data when transformed to inshore values justifies its use for the purpose of gap-filling and extending the wave dataset over the available historical record.

## Additional information

**How to cite this article:** Turner, I. L. *et al.* A multi-decade dataset of monthly beach profile surveys and inshore wave forcing at Narrabeen, Australia. *Sci. Data* 3:160024 doi: 10.1038/sdata.2016.24 (2016).

## Supplementary Material



## Figures and Tables

**Table 1:** Example coastal sites worldwide with ongoing multi-decadal and high resolution coastal monitoring programs (primary source^52^).

**Site**	**Surveys undertaken**	**Example publications**
Duck (USA)	Biweekly beach profiles (1981—)Argus images (1986—)	^1–3^
Rhode Island (USA)	Monthly beach profiles (1962—)	^4^
Egmond aan Zee (The Netherlands)	Annual beach profiles (1964—)Argus images (1998—)Weekly-seasonal beach profiles (2001–2004)	^6–8^
Noordwijk (The Netherlands)	Annual beach profiles (1964—)Argus images (1995—)Monthly 3D dGPS (2001–2004)	^6,9,10^
Lubiatowo (Poland)	Monthly beach profiles (1983—)	^11,12^
Narrabeen-Collaroy (Australia)	Refer Table 2	refer text
Moruya (Australia)	Monthly beach profiles (1972—)	^13,14^
Hasaki (Japan)	Daily beach profiles (1987—)	^15,16^

**Table 2:** Summary of Narrabeen coastal monitoring program (source: ref. 53).

**Survey technique**	**Survey period**	**Survey frequency**	**Spatial coverage**
Historical profile line surveys (Emery method)	April 1976—August 2006	Monthly	Five representative profile lines (subaerial beach)
Historical profile line surveys (RTK-GPS)	May 2005—present	Monthly	Five representative profile lines (subaerial beach)
Argus coastal imaging (Flight Deck Building)	July 2004—present	Hourly (daylight)	Southern half of beach (shoreline mapping)
Argus coastal imaging (Nth Narrabeen/Narrabeen Lagoon)	July 2005—August 2008	Hourly (daylight)	Northernmost 500 m of beach/ Narrabeen Lagoon (shoreline mapping)
RTK-GPS mounted on an All-Terrain-Vehicle	July 2005—present	Monthly	Entire 3.6 km long subaerial beach
Airborne Lidar surveys	July 2011—present	Pre/post major storm events	Entire 3.6 km long subaerial beach and dunes
Permanent fixed Lidar (Flight Deck Building)	May 2014 -present	5 Hz (continuous)	One profile line
Unmanned Aerial Vehicle (UAV) Structure-from-Motion surveys	June 2014—present	Pre/post major storm events	Entire 3.6 km long subaerial beach and dunes
Single/multi-beam hydrographic surveys using boat/jetski (courtesy of NSW Office of Environment and Heritage)	April 2011—present	Irregular	Entire 3.6 km long beach in the surf zone and offshore

**Table 3:** Dataset—monthly cross-shore profile surveys.

**BEACH PROFILE SURVEY DATASET**					
**Profile ID**	**Origin and Orientation [Lat/Long/degN]**	**Data File**	**Time-series**	**Sample Frequency**	**File Format**
PF1	33°42′20.65″S151°18′16.30″E118.42°	Narrabeen_Profiles.csv	27/04/1976 -03/10/2014	Monthly (nominal)	Column 1—Profile IDColumn 2—Survey date (dd/mm/yyyy)Column 3—Chainage (m from origin)Column 4—Elevation (m AHD)Column 5—Flag (‘EMERY’, ‘GPS’, or ‘DUNEFILL’)
PF2	33°42′33.45″S151°18′10.33″E113.36°	as above	as above	as above	as above
PF4	33°43′01.55″S151°17′58.84″E100.26°	as above	as above	as above	as above
PF6	33°43′29.81″S151°17′58.65″E83.65°	as above	as above	as above	as above
PF8	33°43′55.94″S151°18′06.47″E60.48°	as above	as above	as above	as above
(Elevation relative to Australian Height Datum—AHD, corresponding to approximately MSL). Lat/long are referenced to GRS80 Ellipsoid.					

**Table 4:** Dataset–Cross-Shore Bathymetry Transects.

**CROSS-SHORE BATHYMETRY DATASET**					
**Profile ID**	**Origin and Orientation (Lat/Long/degN]**	**Data File**	**Time-series**	**Sample Frequency**	**File Format**
PF1	33°42′20.65″S151°18′16.30″E118.42°	Narrabeen _Bathymetry.csv	07/03/2011–30/07/2015	Irregular	Column 1—Profile IDColumn 2—Survey date (dd/mm/yyyy)Column 3—Chainage (m from origin)Column 4—Elevation (m AHD)Column 5—Bed type (‘SAND’ or ‘REEF’)Column 6—Flag (‘SBEAMJETSKI’, ‘SBEAMBOAT’, or ‘MBEAMBOAT’)
PF2	33°42′33.45″S151°18′10.33″E113.36°	as above	as above	as above	as above
PF4	33°43′01.55″S151°17′58.84″E100.26°	as above	as above	as above	as above
PF6	33°43′29.81″S151°17′58.65″E83.65°	as above	as above	as above	as above
PF8	33°43′55.94″S151°18′06.47″E60.48°	as above	as above	as above	as above
(Elevation relative to Australian Height Datum—AHD, corresponding to approximate MSL, lat/longs are referenced to GRS80 Ellipsoid).					

**Table 5:** Dataset—Inshore Waves.

**INSHORE WAVES DATASET**				
**Profile ID**	**Location (Lat/Long/Depth]**	**Time-series**	**Data File**	**File Format**
PF1	33°42′29.59″S151°18′35.32″E10 m	01/1979–10/2014	Inshore_Waves.csv	Column 1—Profile IDColumn 2—Date and time (AEST, dd/mm/yyyy HH:MM)Column 3—Significant wave height (m)Column 4—Peak wave period (s)Column 5—Wave direction (° TN)Column 6—Flag (‘MEAS’, ‘HIND’ or ‘INTERP’)
PF2	33°42′37.83″S151°18′21.93″E10 m	as above	as above	as above
PF4	33°43′03.68″S151°18′11.73″E10 m	as above	as above	as above
PF6	33°43′28.73″S151°18′12.27″E10 m	as above	as above	as above
PF8	33°43′47.32″S151°18′25.40″E10 m	as above	as above	as above
(Time relative to 24 h Australian Eastern Standard Time—AEST, Lat/longs are referenced to GRS80 Ellipsoid).				

**Table 6:** Dataset—Astronomical Tide.

**ASTRONOMICAL TIDE DATASET**				
**Parameter [unit]**	**Time-series**	**Sample Frequency**	**Data File**	**File Format**
Date & time [dd/mm/yyyy HH:MM]Astronomical Tide [m AHD]	01/1976–10/2014	15 min	Astronomical_Tide.csv	Column 1—Date and time (AEST)Column 2—Astronomical tide (m AHD)
(Time relative to 24 h Australian Eastern Standard Time—AEST. Astronomical tide relative to Australian Height Datum—AHD).				

**Table 7:** Validation of CAWCR wave hindcast for the Sydney waverider buoy.

	**CAWCR WAVE HINDCAST VALIDATION**								
	**Hs**			**Tp**			**Dir**		
**Location**	**R**	**Bias**	**RMSE**	**R**	**Bias**	**RMSE**	**R**	**Bias**	**RMSE**
Offshore	0.90	−0.03 m	0.32 m	0.51	−0.62 s	2.3 s	0.70	−8.4°	30.1°
PF1	0.87	0.01 m	0.25 m	N/A	N/A	N/A	0.69	−3.2°	12.5°
PF2	0.86	0.01 m	0.22 m	N/A	N/A	N/A	0.67	−1.7°	8.7°
PF4	0.84	0.02 m	0.27 m	N/A	N/A	N/A	0.68	−1.8°	9.6°
PF6	0.81	0.04 m	0.28 m	N/A	N/A	N/A	0.69	−2.1°	9.1°
PF8	0.80	0.06 m	0.24 m	N/A	N/A	N/A	0.67	>−1.5°	8.2°

**Table 8:** Validation of SWAN nearshore wave transformation using both measured and hindcast offshore wave data.

	**NEARSHORE WAVE VALIDATION**					
**Offshore Dataset**	**Hs**			**Dir**		
	**R**	**Bias**	**RMSE**	**R**	**Bias**	**RMSE**
Measured	0.94	−0.02 m	0.18 m	0.74	4.4°	11.2°
Hindcast	0.91	−0.04 m	0.22 m	0.62	2.9°	12.8°
